# Implementation and Operational Analysis of an Interactive Intensive Care Unit within a Smart Health Context

**DOI:** 10.3390/s18020389

**Published:** 2018-01-29

**Authors:** Peio Lopez-Iturri, Erik Aguirre, Jesús Daniel Trigo, José Javier Astrain, Leyre Azpilicueta, Luis Serrano, Jesús Villadangos, Francisco Falcone

**Affiliations:** 1 Department of Electrical and Electronic Engineering, Public University of Navarre, 31006 Pamplona, Navarra, Spain; peio.lopez@unavarra.es (P.L.-I.); erik.aguirre@unavarra.es (E.A.); jesusdaniel.trigo@unavarra.es (J.D.T.); lserrano@unavarra.es (L.S.); 2 Institute of Smart Cities, Public University of Navarre, 31006 Pamplona, Navarra, Spain; josej.astrain@unavarra.es (J.J.A.); jesusv@unavarra.es (J.V.); 3 Department of Mathematical Engineering and Computer Science, Public University of Navarre, 31006 Pamplona, Navarra, Spain; 4 School of Engineering and Sciences, Tecnologico de Monterrey, 64849 Monterrey, Mexico; leyre.azpilicueta@itesm.mx

**Keywords:** Intensive Care Unit, visitor control application, hospital, 3D Ray Launching, Smart Health, radio planning

## Abstract

In the context of hospital management and operation, Intensive Care Units (ICU) are one of the most challenging in terms of time responsiveness and criticality, in which adequate resource management and signal processing play a key role in overall system performance. In this work, a context aware Intensive Care Unit is implemented and analyzed to provide scalable signal acquisition capabilities, as well as to provide tracking and access control. Wireless channel analysis is performed by means of hybrid optimized 3D Ray Launching deterministic simulation to assess potential interference impact as well as to provide required coverage/capacity thresholds for employed transceivers. Wireless system operation within the ICU scenario, considering conventional transceiver operation, is feasible in terms of quality of service for the complete scenario. Extensive measurements of overall interference levels have also been carried out, enabling subsequent adequate coverage/capacity estimations, for a set of Zigbee based nodes. Real system operation has been tested, with ad-hoc designed Zigbee wireless motes, employing lightweight communication protocols to minimize energy and bandwidth usage. An ICU information gathering application and software architecture for Visitor Access Control has been implemented, providing monitoring of the Boxes external doors and the identification of visitors via a RFID system. The results enable a solution to provide ICU access control and tracking capabilities previously not exploited, providing a step forward in the implementation of a Smart Health framework.

## 1. Introduction

Sustainability in the provision of health and social services has become one of the main challenges of public administrations in a wide sense. This interest is coupled to the advent of Smart Cities and Smart Regions, in which efficient resource management as well as different governance and data handling models are proposed. Within this context, multiple solutions and studies have been proposed to implement Ambient Assisted Living (AAL) environments to aid elderly persons, people with several degrees of disability or with neurodegenerative diseases [1]; the implementation of Social Sensor networks to aid people in risk of social exclusion [1]; or the combination of e-Health/m-Health services within the context of Smart Cities to provide Smart Health scenarios [2]. In this sense, the introduction of communication systems coupled to medical and social service practice allows monitoring physiological signals which aid in the treatment of chronic diseases in a controlled household environment. In the case of social sensor systems, user behavior, which can also be combined with biomedical signals, provides monitoring capabilities, which enable users to reside in their households whilst guaranteeing normalized living conditions. Field trials have been performed in the case of users with neurological disorders, providing promising results in terms of inclusion [1]. Within the broad health context, hospitals and health centers are a specific use case, given by their size, user density and large variety of activities which take place. Multiple solutions have been adopted to enhance effective hospital management, such as the use and extension of medial health records, automated pharmacy control, patient location and tracking, among others.

ICUs are one of the most demanding scenarios, requiring precise time responsiveness and resource management. ICUs offer particular challenges, such as large number of sensitive equipment, signal acquisition and processing or false alarm handling, among others. Multiple specialized medical equipment can be simultaneously in operation, with different medical specialists requiring access to data, both real time as well as in relation with parameter trends. These data provide relevant information in relation with items such as treatment control, such as sedation, in which adequate treatment doses can be adjusted to the specific patient needs over time. One of the elements that give rise to operational issues is the presence of false alarms, due to inadequate signal interpretation. Not only technical challenges arise, but also logistical elements, such as adequate space usage, logistics of disposable material, accompanying person access and control or unauthorized user access also have to be dealt with. This holds especially true in the case of visitor access control, fundamental in patient recovery process, but usually overlooked on premises access. Studies have been performed to enhance patient control in sedation by using fuzzy logic control systems [3] or by using expert systems in the control of cardiopulmonary disease [4]. Signal processing and integrity is also a relevant issue, especially in order to control the appearance of false alarms [5], for example in the case of ECG signal detection in the case of arrhythmias [6,7], or arterial blood pressure signals [8]. Enhanced performance has also been achieved by employing video signal analysis, in the case of neonatal ICU [9], as well as by processing of audio alarm signals within a similar scenario [10]. Wireless sensor network integration has also been studied in ICU scenarios, with the aim of controlling patient location and position, employing bed weight sensors coupled to wireless transceivers [11].

In this work, an interactive ICU environment is implemented by integrating a Wireless Sensor Network (WSN), in which tracking capabilities and signal I/O acquisition and processing is enabled. One of the main issues in this context is information integrity, given by potential interference values as well by strict coverage/capacity relations (i.e., coverage distance between transceivers in which the required transmission rate is maintained). Therefore, it is compulsory to achieve the required signal levels above transceiver sensitivity thresholds in relation with interference background levels. Precise wireless channel modeling is performed for the specific ICU conditions with the aid of deterministic in-house 3D Ray Launching algorithm, as well as an extensive set of interference channel measurements. Wireless system analysis leads to system configuration, which is tested under real site conditions at the ICU of “Hospital Complex of Navarra” (HCN), in Spain. A set of wireless sensor motes have been employed, as well as specific ICU management application software, in order to provide real time monitoring applications. The rest of the paper is organized as follows: Section 2 presents the ICU context and employed simulation techniques. Section 3 presents simulation as well as measurement results for the channel analysis. Once wireless channel estimations, in terms of coverage values and interference characterization, have been performed, the proposed ICU’s Visitor Access Application and its functionalities are presented in Section 4. Final comments and concluding remarks complete this work.

## 2. Materials and Methods

### 2.1. Scenario under Test: Intensive Care Units (ICU)

#### 2.1.1. Brief Overview of ICUs

An ICU is a healthcare facility that provides intensive medicine. Patients admitted to ICUs suffer from life-threatening—albeit potentially recoverable—conditions. Thus, they require continuous observation, monitoring and support from multidisciplinary specialists as well as a variety of medications and state-of-the-art medical equipment. Since ICUs are highly specialized and technological facilities, they must meet several functional, structural and organizational requirements so that security, quality and efficiency terms are guaranteed.

In a typical ICU, patients are usually allocated in a single ward with direct visual monitoring by the staff by means of a piece of glass, although closed circuit television is also possible. ICUs typically enable voice intercommunication between the ward and the monitoring zone. In addition, specific alarm systems for critical situations (e.g., cardiorespiratory arrest) are usually installed.

ICU-admitted patients, given their critically illness, require the monitoring of a number of vital signs, such as heart rate, blood pressure, respiratory rate, pulse oximetry, hourly urine output, temperature, or blood gases [12]. Generally speaking, intensive care is suitable for patients requiring support of one or more organs, being the lung the most commonly treated. Besides respiratory support, patients in critical care usually require support in body systems such as circulatory, renal, neurological and metabolic systems [13]. To provide such support, the medical equipment normally included in an ICU comprises a variety of devices, such as ventilators (for respiratory support), hemofiltration or hemodialysis equipment (for renal support), multi-parameter monitors (which continuously monitor bodily parameters such as electrocardiogram, heart rate, temperature, blood pressure, pulse oximetry, end-tidal CO_2_, oxygen saturation, cardiac output, etc.), infusion pumps (for infusing fluids, medication or nutrients into the circulatory system), among other equipment [14,15].

Besides the patient, there are two major groups involved that are worth discussing here: the staff and the visitors. Concerning the staff, a multidisciplinary team—with a high staffing-to-patient ratio (i.e., with a high number of staff people vs. the number of patients)—is usually in charge of providing critical care. The intensivist is the foremost specialized physician, who is an expert in the care of critically ill patients. Besides intensivists, other specialists may contribute to ICUs, such as anesthetists, radiologists, infectologists, pathologists, surgeons, or neurologists. Specialized nurses work alongside the specialists. Since ICU nurses provide continued care for the patients, their work is of utmost importance. They monitor and care about the wellbeing of the patients and must be competent with the technical procedures and devices used in the ICU. Indeed, nurses’ attitudes and perceptions of new technologies are strategic to successful implementation. Lastly, other healthcare professionals are usually involved in ICUs, e.g., physiotherapists, pharmacists, dietitians, and microbiologists, as well as other professionals, such as technicians, hospital porters, or cleaning staff [13,15,16].

Visitors play a crucial part in the patient recovery process. Traditionally, visits were widely restricted under the premises of higher risk of infection, which is better controlled today; the patient’s need for rest and quietness; and the presumption that visitors may interfere with medical staff work. However, ICUs can be vastly distressing and the presence of relatives can be regarded as positive, since relatives can help in encouraging physical and intellectual stimulation as well as in providing adequate psychological an emotional support [16]. Thus, current philosophy tends to open visit policies [17], but it is still limited in practice and should be implemented with caution [18].

#### 2.1.2. ICU of the “Hospital Complex of Navarra” (HCN)

**Figure 1:**
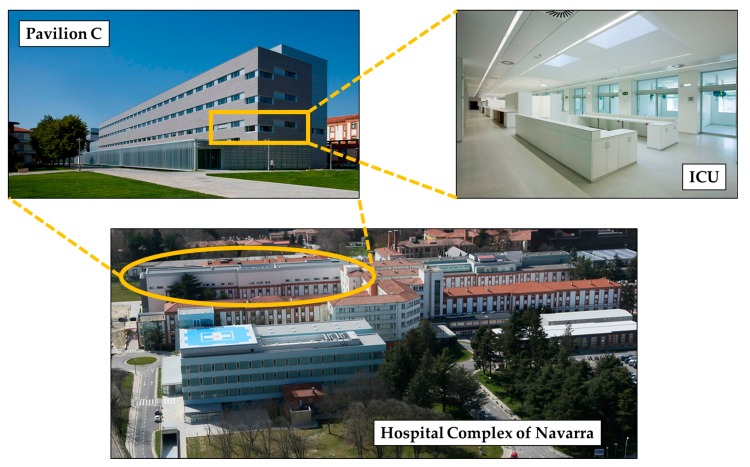
Scenario under analysis: the ICU-A of the HCN, located in Pamplona/Iruña, Navarre, Spain.

This paper is focused on the ICU of the HCN located in Pamplona/Iruña (Spain). According to Navarra’s government data, 1200 patients are admitted yearly into the ICU. The unit was remodeled in 2015 and is further divided into two ICU facilities: ICU-A and ICU-B. The analysis provided in this paper is focused in ICU-A, which comprises 24 beds, one within each of the 24 Boxes. Figure 1 shows the scenario.

**Figure 2:**
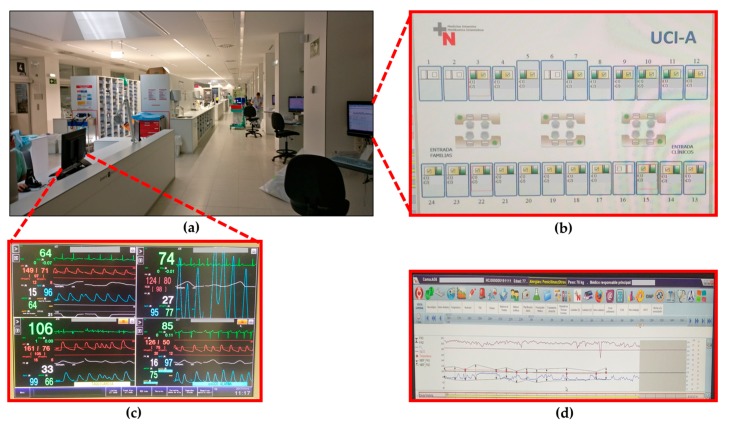
Screenshot of different tools of the management software of ICU-A: (**a**) ICU-A; (**b**) Boxes occupation tool; (**c**) Vital signs graphs; and (**d**) Monitored parameters screenshot.

A wide range of medical equipment is allocated in each Box of the ICU-A, as described in Table 1. The devices with communication capabilities gather the medical data to the information system, so that every pre-configured parameter is stored and a graph is generated for the nursing staff. It is important to note that all communications are wired. All collected data are managed with the aid of specific software developed for the ICU-A. Figure 2 shows some of the most relevant screenshots of the management software, which can be accessed from all the computers within the ICU-A. In Figure 2b, the occupation of the Boxes is shown graphically. As can be seen, 20 out of the 24 Boxes are occupied (those in green and yellow), while four beds are free (Boxes 1, 2, 6 and 16). In Figure 2d, a screenshot of the parameters monitored and controlled by the medical staff can be seen. Additionally, nurses at the central zone control all the multi-parameter monitors that are sending the vital signs and handle the potential alarms (see Figure 2c). The names of patients and physicians have been redacted from all the screenshots.

Regarding the visiting policy, the ICUs of the HCN have embraced an open visiting policy, aligned with current trends. However, as an excessive number of visitors in a specific Box might be counter-productive, a recommendation of maximum two visiting persons at the same time for each Box is displayed on posters. At this time, there is no method for controlling or monitoring access, and nursing staff take over this purely managerial function. Thus, there is an opportunity to propose and provide new automatized wireless services for such purposes, so that nurses have more time to engage with their medical-related tasks.

### 2.2. Ray Launching Simulation Technique

#### 2.2.1. Background

In this subsection, the simulation technique used for the radio planning tasks previous to the deployment of a WSN is presented. In general, hospital environments are very complex in terms of morphology, interference and variability of human being and equipment presence. It has been reported in the literature that wave propagation in hospitals and health environments is different from other buildings, due to the special construction of the walls. These walls contain metallic layers within their structure, e.g., operating rooms, X-ray rooms or magnetic resonance rooms, with lead or copper shielded walls. The radio channel characterization in such rooms is a key factor to assess and prevent EMC (Electromagnetic Compatibility) between different medical devices. In the same way, it is important to analyze the wave propagation to design an efficient use of wireless devices, even more when they are deployed around critical care medical equipment, where their immunity to electric field strength has to be taken into account.

There are some works in the literature that analyze wave propagation in hospital environments using different methodologies. The first approach comprehends empirical or statistical methods, which are based on measurements. The main advantage of these methods is that they finish quickly. However, they require adjustments based on in-situ measurements to give a good fit of the estimated results. In [19], the radio channel model for very high-speed radio systems (60 GHz) in hospital environments is presented, specifically with two possible applications: real time video streaming and angiography and ultrasonic imaging. The channel model parameters are described statistically and implementation guidelines in hospital environments are given in the work based on measurements. A novel statistical path loss model based on measurements taking into account the variability of height and the attenuation caused by the human beings at both frequencies, 6 and 8.5 GHz, under a hospital scenario is presented in [20]. It is shown that the proposed model is more flexible and accurate when compared with equivalent methodologies.

However, these methods do not consider all the elements in the environment. Thus, they can give erroneous results on the electromagnetic propagation estimation when the morphology of the scenario has a significant impact (due to its complexity), such as hospital environments, which usually have a complex structure of the walls, different type of scatterers, materials, mobile equipment, etc. Thus, the most widely used approach for propagation prediction in this type of scenarios corresponds to deterministic methods. The main principle of these methods is based on ray optics with advanced ray tracing techniques, or on solving Maxwell’s equations. These methods are accurate, but are also time-consuming due to inherent computational complexity. Methods based on geometrical optics (GO), such as ray launching (RL) or ray tracing (RT), offer a reasonable trade-off between precision and required calculation time [21]. The difference between RL and RT is that, in the RL technique, rays are launched from a transmitter, and when they intersect with an obstacle, the new reflected, absorbed, diffracted, or scattered rays are created, while, in RT techniques, imaging techniques are usually employed, creating several possible paths that rays follow from the transmitter to receiver over the direct, reflect and diffracted rays. In [22], a ray tracing approach which considers information about intersection techniques and the hither neighbor cell is presented to characterize wave propagation between the control units (CUs) and Wireless Access point (AP) in a hospital scenario, showing the effects on received power in 60 GHz frequency. In reference [23], the simulation of 60 GHz radio channel in a hospital ultrasonic inspection room using ray tracing methodologies with a single reflection scattering is presented, showing a good match with measurements. In [24], the characterization of radio wave propagation in hospitals in the frequency range from 42.6 MHz to 5.2 GHz is presented. They use a simulation tool based on the finite-difference time-domain (FDTD) for the lower frequencies, and a ray optical tool for higher frequencies up to 5.2 GHz. They conclude that wall attenuation in hospital areas is not the usual because of the different metallic layers in their structure.

#### 2.2.2. The Ray Launching Technique

Following the trend, in this work, an in-house developed three-dimensional (3D) RL tool has been used for the channel characterization of the Intensive Care Unit (ICU) area of the previously presented HCN. The proposed in-house 3D RL algorithm has been previously used and validated in large complex indoor environments [25,26,27], and also specifically in a hospital environment [28]. The algorithm is based on GO and the Uniform Theory of Diffraction (UTD). The principal basis of the algorithm is that rays are launched from the transmitter with a determined angular and spatial resolution. When a ray impacts an object, both reflected and refracted rays are created, and, when a ray hits an edge, a new family of diffracted rays is created. Electromagnetic phenomena such as reflection, refraction and diffraction are taken into account, as well as all the material properties of all the obstacles within the environment, considering its conductivity and relative permittivity at the frequency of the wireless system under analysis. Hybrid techniques can also be used with this in-house developed RL tool, depending on the dimensions and the complexity of the considered scenario. Different modules have been created in the RL tool, such as the Neural Network (NN) module [29], the Diffusion Equation (DE) approach [30], or Collaborative Filtering (CF) module [31]. These novel hybrid techniques can be used with the RL tool, leading to accurate results while computational burden decreases significantly.

**Figure 3:**
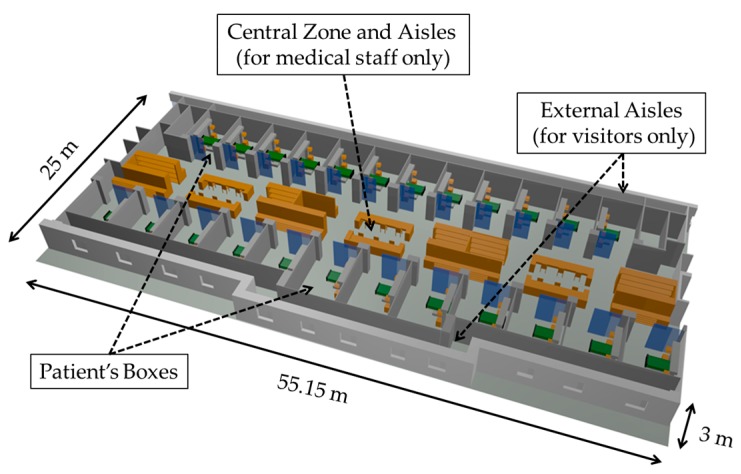
UCI-A scenario created for its simulation with the in-house 3D Ray Launching algorithm.

The complete area of the UCI-A has been created for its simulation with the 3D Ray Launching tool. The schematic view of the created scenario can be seen in Figure 3, where the central zone (for the medical staff), Boxes for patients and the external aisles for the visitors are indicated. For its creation, all the dimensions and main furniture’s size have been obtained from the CAD model of the building. The material properties of furniture, beds, walls, floor, ceiling and Boxes’ equipment have been selected as close as possible to the real ones to obtain accurate simulation results. Conductivity and electric permittivity of all the obstacles within the environment have been considered at the frequency under analysis, as input parameters of the algorithm. The principle of the 3D RL algorithm is that rays are launched from the transmitter with an angular resolution of rays of θ and ϕ as an input parameter. The scenario can be divided into a fixed number of cuboids with different dimensions in X, Y and Z axes. Thus, when rays are launched from the transmitter, each of them follows a different path and all the parameters associated with each ray are stored in each cuboid that it goes through, calculating also the distance traveled by each ray. When a ray hits an object, new reflected and refracted rays are created, following the principle of Snell’s law, always taking into account the material properties of the object. In addition, when a ray hits an edge, a new family of diffracted rays are created, following diffraction laws (UTD).

## 3. Results

This section has been divided in two subsections. First, with the aim of studying the availability of wireless communication frequency bands within the ICU, an assessment in terms of RF (Radio Frequency) noise is presented. Then, before the deployment of the real devices, radio planning tasks regarding the proposed WSN are carried out to obtain information about its adequacy.

### 3.1. RF Assessment of the Environment

**Figure 4:**
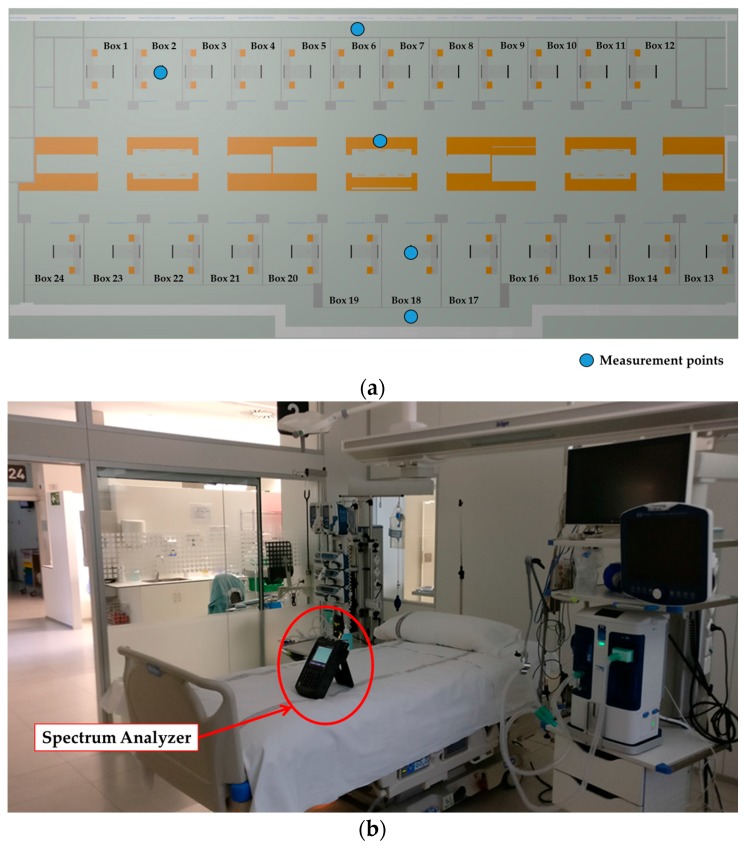
(**a**) Points within the scenario where spectrograms have been measured; and (**b**) the employed FieldFox N9912A spectrum analyzer of brand Agilent (Las Rozas, Spain) within Box 2.

**Figure 5:**
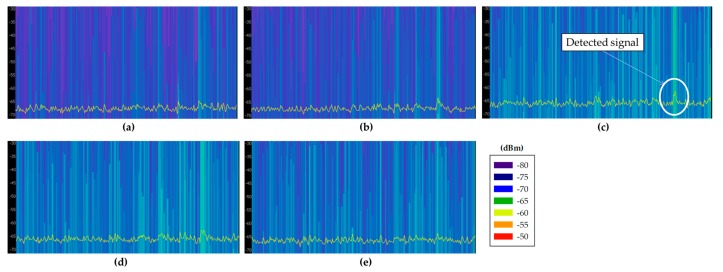
Measured spectrograms at 433 MHz central frequency with 20 MHz bandwidth: (**a**) within Box 2; (**b**) within Box 18; (**c**) aisle in front of Box 6; (**d**) aisle in front of Box 18; and (**e**) in the middle of the scenario.

**Figure 6:**
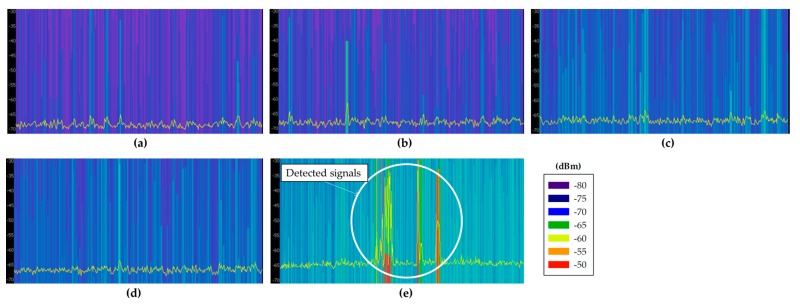
Measured spectrograms at 868 MHz central frequency with 30 MHz bandwidth: (**a**) within Box 2; (**b**) within Box 18; (**c**) aisle in front of Box 6; (**d**) aisle in front of Box 18; and (**e**) in the middle of the scenario (bandwidth of 300 MHz).

**Figure 7:**
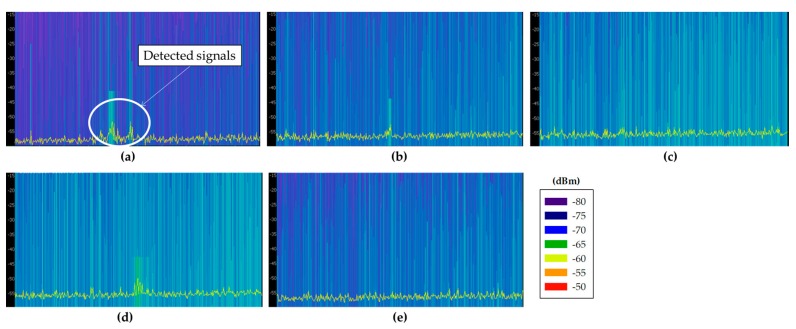
Measured spectrograms at 2.45 GHz central frequency with 100 MHz bandwidth: (**a**) within Box 2; (**b**) within Box 18; (**c**) aisle in front of Box 6; (**d**) aisle in front of Box 18; and (**e**) in the middle of the scenario.

**Figure 8:**
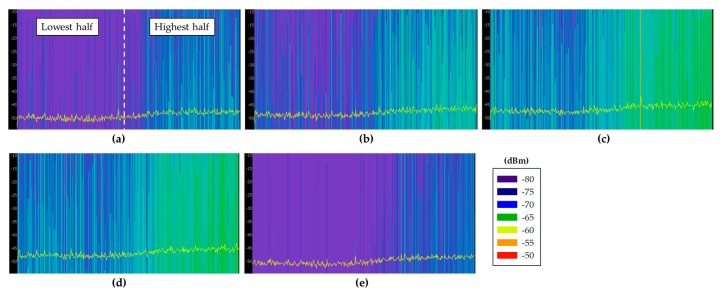
Measured spectrograms at 5.5 GHz center frequency with 600 MHz bandwidth: (**a**) within Box 2; (**b**) within Box 18; (**c**) aisle in front of Box 6; (**d**) aisle in front of Box 18; and (**e**) in the middle of the scenario.

Before choosing an adequate wireless communication technology for the deployment of the system, radio planning tasks have been carried out within the scenario. Specifically, background noise measurements have been performed at different typical communication frequencies where medical devices can operate, such as 433 MHz [32], 868 MHz [33], 2.4 GHz [33] and 5 GHz [34]. The aim of these measurements was to analyze the existence of potential interferences at the mentioned frequency bands, since the coexistence of wireless medical devices is an important factor for the deployment of new wireless networks [35]. For that purpose, different antennas have been used, connected to a FieldFox N9912A portable spectrum analyzer. The characteristics of the employed antennas are summarized in Table 2. Specifically, spectrograms have been measured on each point, as they provide information of the power level of the received signal within a specified frequency bandwidth during a time interval, which for these measurements was of 5 min. The measurements have been taken in different zones of the scenario: within boxes 2 and 6 (since there were no patients inside), on a table of the central zone and in the middle of the external aisles. All these measurement points are represented by blue dots in Figure 4a, whilst Figure 4b shows the employed spectrum analyzer within Box 2. Figure 5 shows all the measured spectrograms at 433 MHz band, Figure 6 at 868 MHz, Figure 7 at 2.4 GHz and Figure 8 at 5 GHz band. The shown bandwidths and central frequencies correspond to the data in Table 2.

As can be seen in the spectrograms, the analyzed communication bands are almost interference free except for the 868 MHz band. The 433 MHz band presents a signal around 440 MHz in all the measured spectrograms, but the rest of the band is free. On the other hand, the 868 MHz band is the most interfered band measured in this scenario. Figure 6e shows different signals with a high power level (i.e., similar to radio communication signal levels), which could greatly interfere a wireless communication. Note that the shown bandwidth of Figure 6e has been increased to 300 MHz to capture the whole interference. Due to its pattern, these detected signals are probably produced by an operating medical device. The 2.4 GHz band is almost signal free mainly because there is no WiFi access point on the whole floor of the building. Regardless, some signals can be seen at the center of the band, which probably are WiFi signals coming from WiFi access points outside this floor of the building or from the mobile devices of the medical staff present within the scenario when the measurements were carried out. Last, the 5 GHz band seems to be interference free. The background noise level difference that can be seen between the lowest half of the spectrum and the highest half is due to the employed antenna, which has not the same gain for all the measured bandwidth. Finally, it is worth noting how the detected background noise levels for all the frequency bands are higher for the measurements taken within the external aisles. This effect can be seen in all (c) and (d) spectrograms of Figure 5, Figure 6, Figure 7 and Figure 8, which presents higher background noise level than the rest of measurement points. In summary, the closer to the street, the higher is the background noise level.

**Figure 9:**
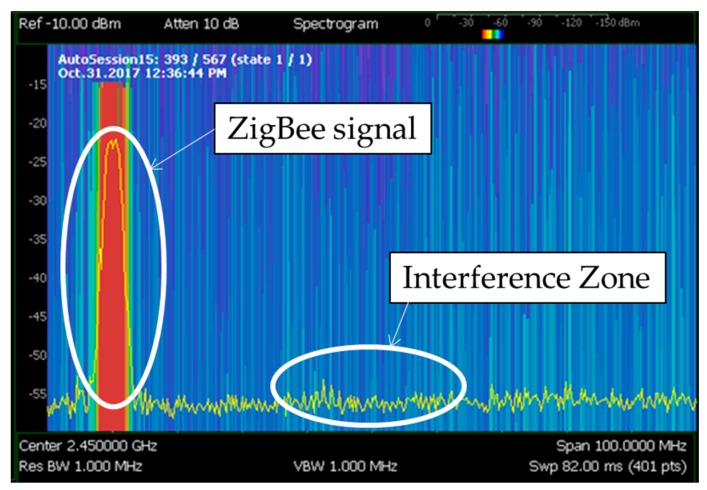
Measured spectrogram at 2.4 GHz band in the middle of the scenario with the XBee mote transmitting 10 dBm at ZigBee channel C (2.41 GHz).

Taking into account these results, the 868 MHz band was discarded immediately as operation frequency band due to the high level of interference that can be found within the scenario. The rest of the measured bands are almost completely available for the deployment of wireless devices. However, among 433 MHz, 2.4 GHz and 5 GHz bands, the band of 2.4 GHz band has been chosen due to the advantages that provides and the great amount of devices that are available in the market. Specifically, ZigBee-based devices have been chosen in order to deploy the WSN within the ICU due to its low energy consumption (mainly due to the low transmission data rate of 250 kbps), high reliability in terms of the transmitting packets routing (auto-configurable mesh topology, up to 3 retransmissions by the use of ACKs and CSMA-Collision Avoidance) and the relatively low cost in terms of device cost and license free frequency band (ISM 2.4 GHz). Besides, as ZigBee’s physical layer is based on the standard IEEE 802.15.4, it provides immunity to in-band interference and multipath propagation [36]. In the same way, there are 16 frequency channels in the 2.4 GHz band available, which give the possibility to avoid unexpected interferences by changing the transmission channel. In fact, as mentioned previously, Figure 7 shows some signals detected around 2.45 GHz. Therefore, ZigBee channel C, which corresponds to 2.41 GHz central frequency channel, has been selected to operate within the scenario, avoiding this potential interference at 2.45 GHz. In fact, most of the channels could be used, as they do not either overlap the interference. To show this graphically, Figure 9 shows a spectrogram measured in the middle of the scenario with a ZigBee-based XBee mote transmitting 10 dBm at ZigBee channel C. As can be seen, the frequency channel does not overlap with the 2.45 GHz potential interference. It is important to note that the measurement of Figure 9 has been made with the ZigBee mote just beside the spectrum analyzer, which is the reason of why the detected power is high.

### 3.2. Ray Launching Simulation Results

**Figure 10:**
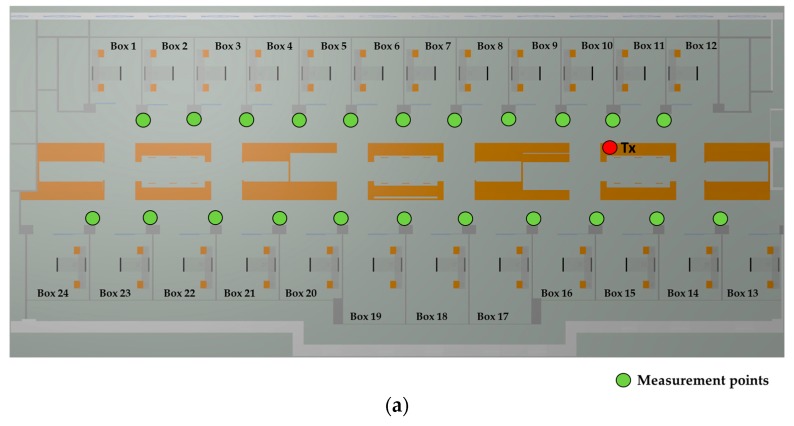

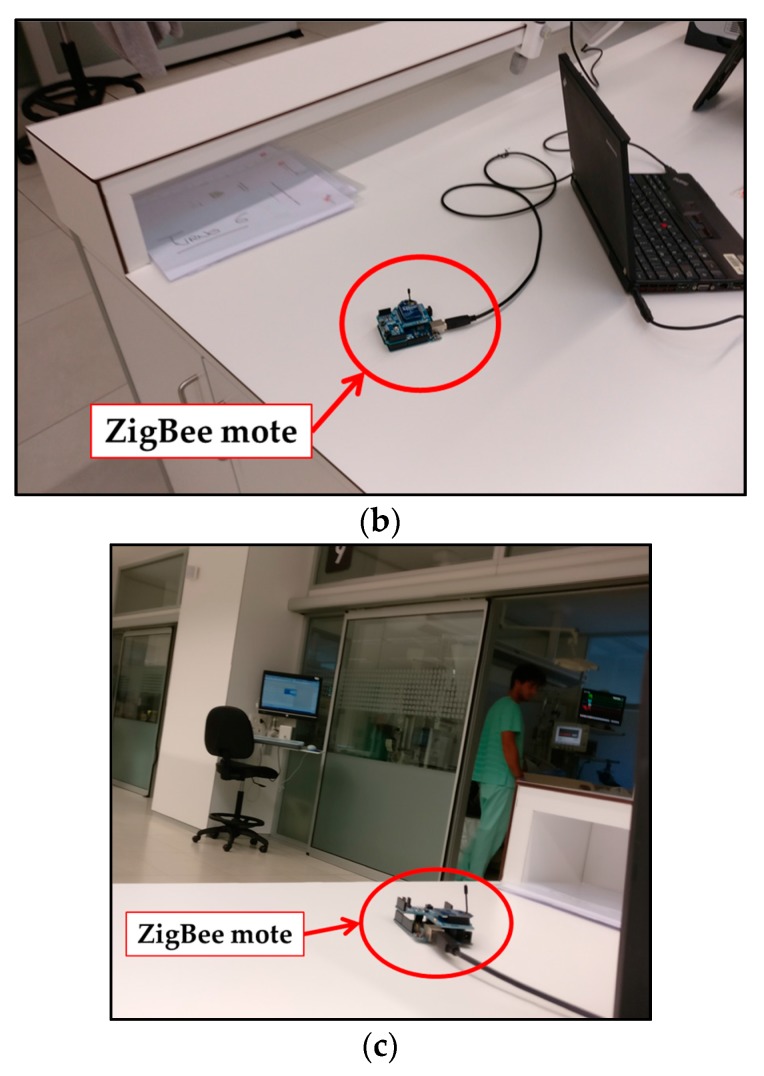
(**a**) Schematic view of the scenario with the position of the transmitter (red dot) and the measurement points (green dots); and (**b**,**c**) the detail of how the XBee-Pro mote has been deployed.

Once the wireless technology and the operating frequency channel for the deployment of the WSN have been selected, a new measurement campaign has been carried out in order to validate the presented 3D Ray Launching simulation tool for its use in this scenario. For that purpose, a Zigbee-based mote (XBee-Pro mote) connected to a laptop via USB cable has been placed on one of the tables used by the medical staff, and several received power level measurements have been taken along the internal aisles of the scenario. Figure 10a shows the position of the transmitter (represented by a red dot) and the measurements points (represented by green dots). Figure 10b,c shows how the transmitter has been deployed. The mote has been configured to operate at 2.41 GHz channel with its maximum transmitted power level of 18 dBm. For the received power level measurements, the corresponding antenna (see the 2.45 GHz antenna of Table 2) connected to the Agilent FieldFox N9912A spectrum analyzer has been used, the same way that can be seen in Figure 4b. All measurements have been taken at 1.5 m height.

**Figure 11:**
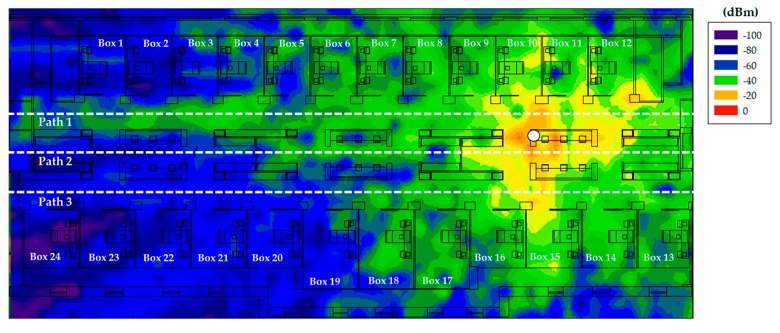
RF power distribution at height 1.5 m obtained by the 3D Ray Launching software. Transmitter is represented by a white dot.

**Figure 12:**
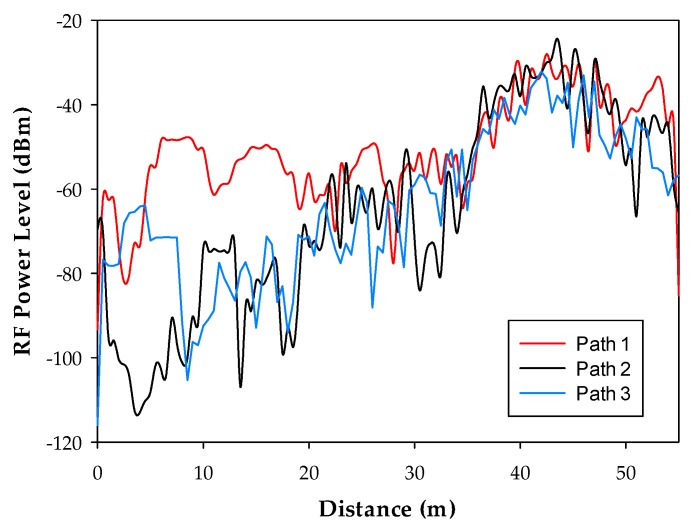
Three paths for linear RF power level distribution. The results correspond to the white dashed lines in Figure 11.

**Figure 13:**
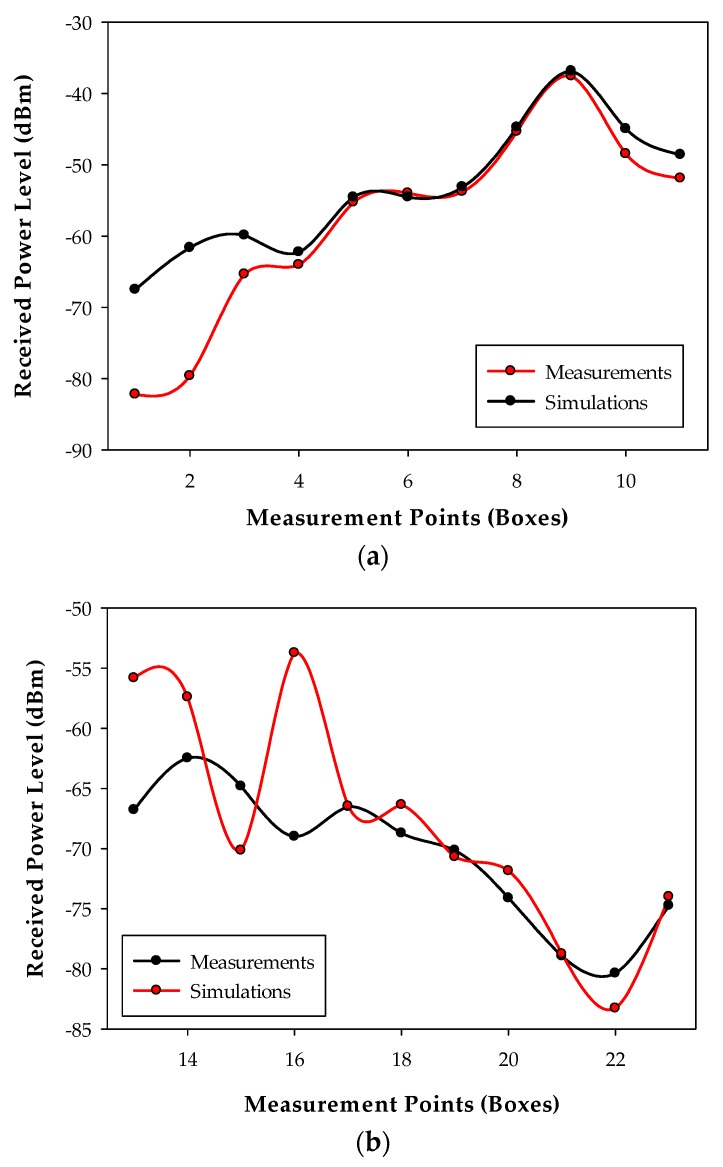
Measurements vs. 3D Ray Launching simulation results: (**a**) for Boxes 1–12; and (**b**) for boxes 13–24.

**Figure 14:**
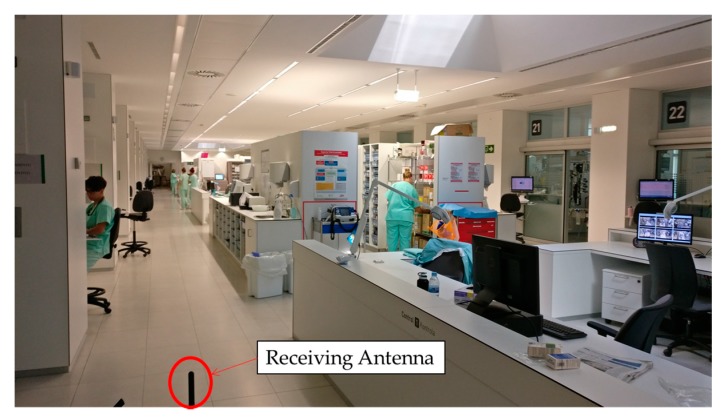
Picture of the UCI, taken during the measurements.

Once the measurements have been taken, the 3D Ray Launching simulation has been launched in order to obtain the estimated RF power distribution for the whole volume of the scenario. Table 3 shows the main parameters used for the 3D Ray Launching simulations, which have been selected as close as possible to the real parameters of the XBee-Pro module and the antenna of the spectrum analyzer. Figure 11 shows a bi-dimensional plane of the RF power distribution at height 1.5 m. Generally, in indoor complex environments, the most important radio propagation phenomenon is the multipath propagation. Due to the complex morphology of this scenario, the multipath propagation has a great impact on the radio propagation. This phenomenon causes rapid RF power variations along the distance. To see this effect, in Figure 12 three linear paths vs. received power level graphs are shown. These curves correspond to the three white dashed lines in Figure 11. The typical rapid RF power variations due to the multipath propagation can be clearly noticed in Figure 12. Now, the estimated received power level at the same positions of the measurement points in Figure 10a can be obtained to validate the simulation tool. Figure 13 shows the comparison between the measurements and the simulation results. The measurement points are numbered from 1 to 23, and each number refers to the point between two Boxes, e.g., measurement point number 1 refers to the green point in Figure 10a placed between Box 1 and Box 2, and so on. As can be seen in the comparison graph, for most of the points, the estimated values are very close to the measured ones, but there are a few points where the difference is quite high (more than 10 dB in some cases). These high error points are due to the different conditions between the simulated scenario and the real scenario: On the one hand, when the measurements were taken, the medical staff was working and constantly moving throughout the ICU (as can be seen in Figure 14), and there was some mobile equipment, such as X-ray equipment, which was moved throughout the scenario. On the other hand, simulations have been performed without this extra equipment and without the presence of human beings, which could have a great impact in terms of received power level [37]. Nevertheless, the 3D Ray Launching simulation tool can be considered validated as the mean error taking into account the measurements at the 22 points shown in Figure 10a and their corresponding simulation estimations (see Figure 13) is 3.49 dB with a standard deviation of 5.86 dB.

After the validation of the 3D Ray Launching tool, the assessment of a proposed ZigBee-based WSN deployment for the proposed application for the ICU is discussed next. As the WSN devices are going to be deployed on each of the external doors of all the boxes (as can be seen in Section 4), there is no need to find the best location for the transceivers. However, it is important to estimate if the proposed WSN will perform satisfactorily and optimally before its deployment, as the ICU is a scenario where less time and fewer tests are better for the patients and the medical staff. Therefore, new simulations have been performed to analyze the real positions of the transmitted elements within the scenario, i.e., placed on the external doors of the Boxes.

**Figure 15:**
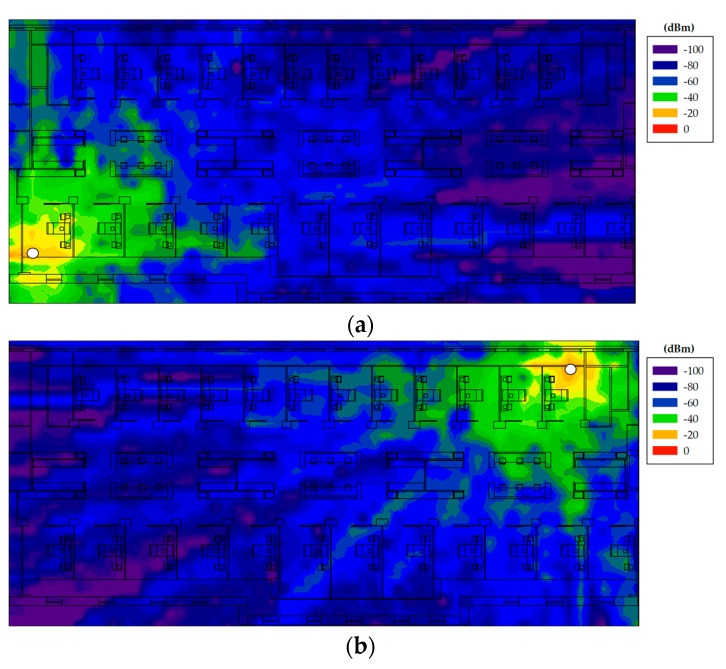
RF power distribution obtained by the 3D Ray Launching tool for two different locations of the proposed ZigBee-based WSN. The white dots represent the ZigBee devices on the external doors of Boxes 12 and 24.

**Figure 16:**
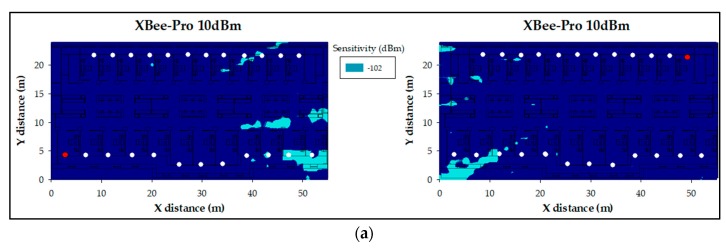

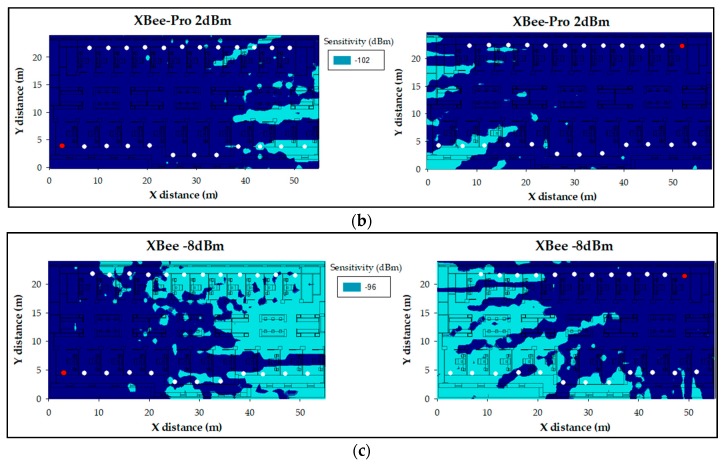
Sensitivity fulfillment planes corresponding to: (**a**) XBee-Pro motes transmitting 10 dBm; (**b**) XBee-Pro motes transmitting 2 dBm; and (**c**) XBee motes transmitting −8 dBm. Red dots represent the transmitter mote position.

In Figure 15, the obtained estimations by means of the 3D Ray Launching algorithm can be seen. As an example, two specific positions of ZigBee transmitters are shown. The simulation parameters are the same of those shown in Table 3 but the transmitted power level, which has been set to 10 dBm (i.e., the maximum power level for the International version of ZigBee XBee-Pro modules). A key condition that must be fulfilled at receiver locations is to exceed the sensitivity value of the wireless modules, which in this case is −102 dBm. This threshold must be fulfilled at the receiver in order to be able to communicate with the transmitter. With the aim of showing this graphically, Figure 16a shows the sensitivity fulfillment planes corresponding to the results of Figure 15. As can be seen, the sensitivity is fulfilled for almost the whole scenario (at the presented height).

However, there are other important aspects that must be taken into account, such as the power consumption of the wireless motes. As said, the transmitted power level has been firstly set to the maximum (10 dBm). However, since the sensitivity criteria are exceedingly fulfilled, new simulations have been performed setting the transmitted power to the minimum of the motes, i.e., 2 dBm. As can be seen in Figure 16b, the sensitivity fulfillment zone (dark blue) diminished significantly since the overall RF power within the scenario decreases. Nevertheless, there are still many ZigBee motes’ locations that comply with the sensitivity threshold. Taking into account that a ZigBee network (operating in mesh topology) could route automatically the received packets to reach the net coordinator, it could be considered to transmit less power, even more taking into account that the used frequency channels are interference free.

Therefore, alternative motes have been analyzed and used for the deployment of the WSN, since the XBee-Pro modules’ transmitted power cannot be less than 2 dBm. Specifically, the XBee motes have been chosen. These motes are a variant of the XBee-Pro modules which the main difference with the previous ones is that they are smaller, cheaper, they have a slightly worse sensitivity level (−96 dBm) and their transmitted power range goes between 2 dBm and −8 dBm. Now, simulations with the lowest value of −8 dBm have been performed, and the results in terms of sensitivity can be seen in Figure 16c. The sensitivity fulfillment has been drastically reduced, but the RF power distribution is still enough to communicate with other XBee motes of the WSN. Therefore, the ZigBee XBee motes have been chosen to deploy the wireless network as they will consume less energy (lower transmitted power) and the cost of the transceiver is lower.

A summary of all these performed simulations is shown in Table 4, which corresponds to the results shown in Figure 16.

## 4. Visitor Access Control Application

As previously mentioned, ICU visitors play a crucial part in the recovery of patients, but, at the same time, the current trend of open visits should be implemented with caution. In the ICU under analysis in this study, the access of visitors is made from the core of the hospital’s external circulation. The visitors are allowed only at the preset visiting hours (except in specific cases), and the access to patients is via the Box doors of the external aisles. When visiting hours are finished, the access to external aisles is closed, and, therefore, the access to the Boxes. However, in this ICU, the access to the external aisles is out of reach of direct visual control from the reception area of the unit. Besides, the external doors of the Boxes have a simple manual lock, which can be easily leave open unintentionally by medical staff or cleaning staff. Thus, the patient’s health could be put at risk (e.g., for isolated patients), and the security of the ICU itself could be compromised.

On the other hand, the current “open visit” policy recommends that only one or two companions could remain with the patient for as many hours as possible in order to provide psychological and emotional support to the patient. In any case, the hospital must ensure that the privacy and safety of patients is always preserved. For such reasons, ICUs need to define mobility limitation measures, and also to implement access control and monitoring mechanisms. Therefore, a Visitor Access Control Application has been developed in this study.

**Figure 17:**
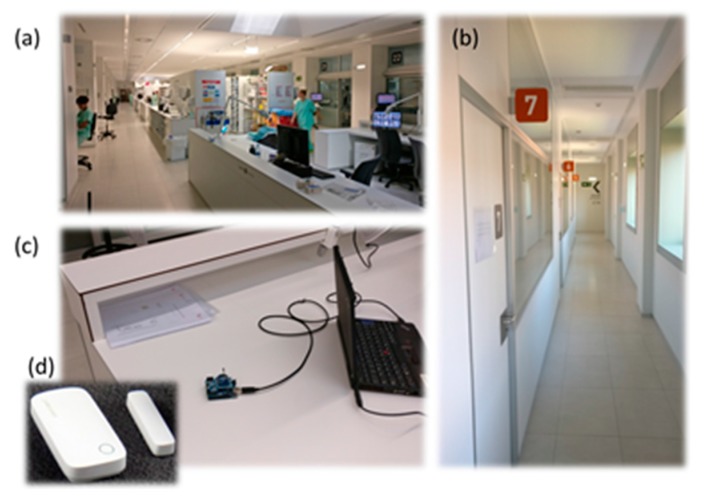
Working scenario: (**a**) central Zone; and (**b**) external aisles; and used wireless devices: (**c**) remote control device; and (**d**) open/close sensors.

The application consists on the monitoring of the Boxes external doors and the identification of visitors via a RFID system. For the monitoring of the Boxes external doors, a ZigBee-based implementation has been selected, as seen previously. Figure 17 shows the working scenario as well as the external aisles, the remote control device (the ZigBee coordinator) and the used open/close sensors. Each external door of the ICU care Boxes will have an open/close sensor that notifies to the remote control device the change of the Box door status (opened/closed) via a ZigBee communication. Besides, a RFID reader will permit (or not) the access to the visitor (who should have received a RFID tag) to the specific Box, thus, each visitor can access only the Box corresponding to his/her relative.

**Figure 18:**
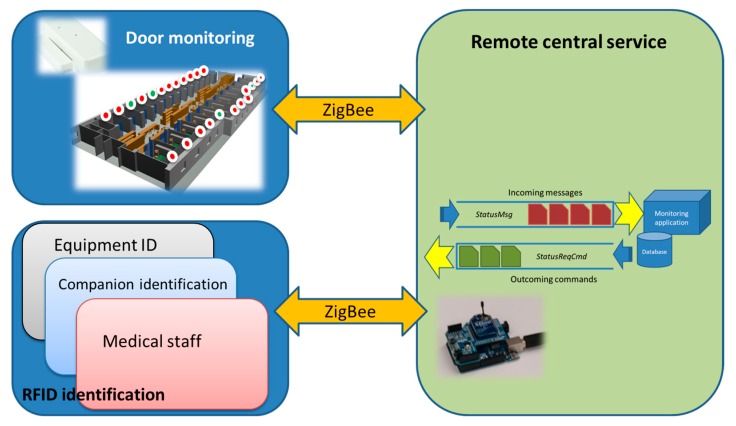
System architecture schema.

**Figure 19:**
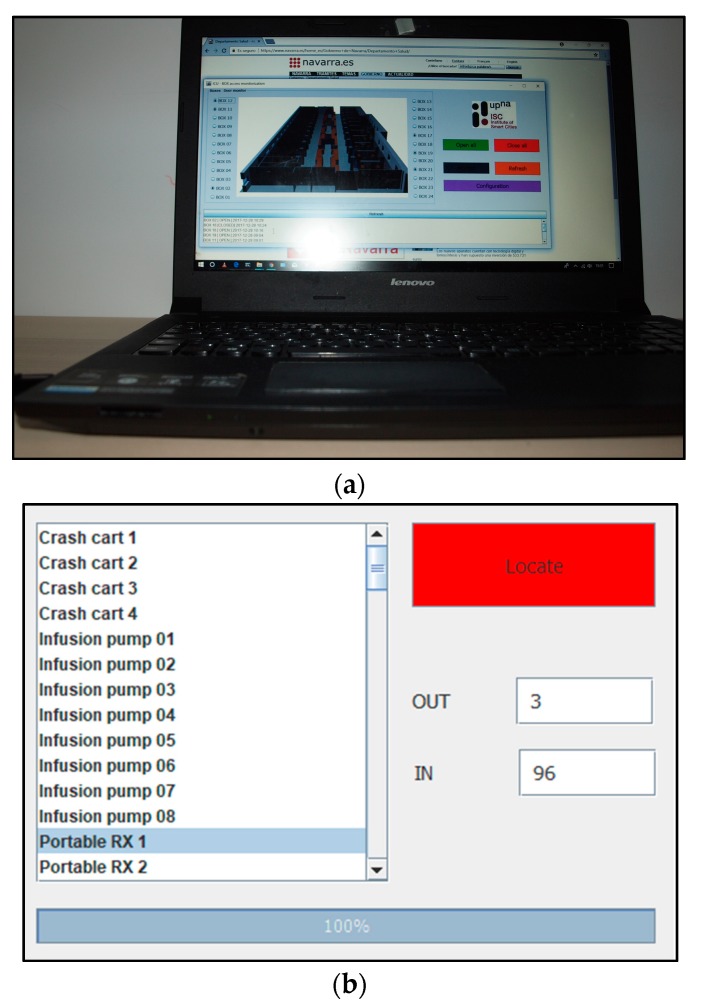

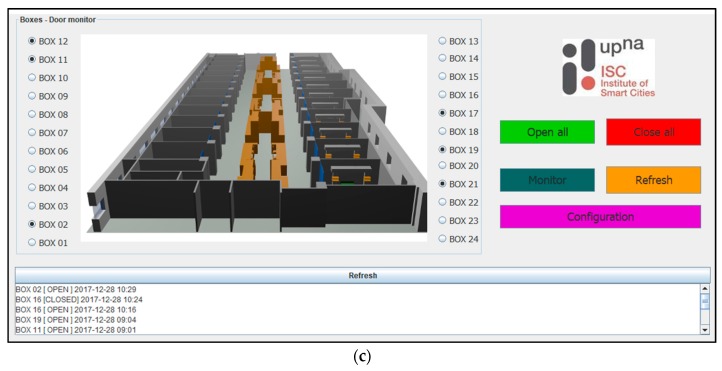
Application interface: (**a**) application running on a laptop; (**b**) equipment location control; and (**c**) boxes door monitoring.

The system uses the lightweight messaging protocol MQTT (Message Queue Telemetry Transport) to minimize data packet size and power consumption. MQTT allows implementing passive (push) and active (pull) monitoring of the doors status as well as efficient distribution of the gathered information. Passive monitoring implies that sensors are themselves responsible for notifying the change of status to the remote central service (i.e., the ZigBee coordinator of the network). Each time a door is opened/closed, the sensor notifies it to the central service without requiring any kind of user intervention. Sensors push the information to the service. On the other hand, active monitoring implies a pull mechanism since it is done on demand. Each time a user (medical staff) needs to know the status of a given door, or a set of them, the central service interrogates the different sensors involved and refreshes the situational awareness. Figure 18 shows the architecture of the proposed monitoring system. The application and the system provide the possibility to extend the access control to medical staff and medical equipment such as portable RX equipment or portable ultrasound scanners, but at this estate of development they have not been implemented. Figure 19 illustrates the application interface.

Finally, it is important to note that the system could be modified (if required) to supply devices with energy harvesting methods (those devices with low energy consumption). In the same way, a greater level of confidentiality can be provided to the wireless communications if messages are encrypted. This can be easily done as the used ZigBee motes have the possibility to activate the cryptography option (in exchange of higher energy consumption and latency increment of the system).

## 5. Discussion

In this work, a context aware ICU environment has been developed, by combining a WSN formed by adapted motes with ad-hoc application software to enable user interaction. Due to the specific nature of ICUs, an extensive wireless channel analysis has been performed in order to gain insight in limitations given by interference, as well as to guarantee coverage/capacity relations, which are given by interference values as well as by signal fading. Precise wireless channel characterization is compulsory in order to consider large signal variations present in complex indoor scenarios, given mainly by multipath propagation components. Deterministic 3D RL simulations provide channel information for the complete volumetric scenario, providing information in relation with WSN transceiver mote location, applicable to a wide range of elements within the ICU, such as instrumentation, material/waste logistics or perimeter tracking and user location and visitors access control. The employed 3D RL code implemented in-house has been optimized by means of convergence analysis in order to optimize simulation parameters (given by angular resolution and number of rays until extinction). Moreover, hybrid simulation based on the combination of 3D RL with Neural Network interpolators, or Collaborative Filtering techniques allow analyzing large volumetric scenarios in a precise manner. In this way, full coverage estimation for the complete scenario volume are obtained, which enable deployment strategies in any potentially required location. On site measurements have been conducted at typical WSN operational frequencies (433 MHz, 868 MHz, 2.4 GHz and 5 GHz), in which interference analysis indicates that their operation is feasible. Time/frequency spectrograms have been obtained from the measurement campaign done within the ICU, in which interference conditions, which can be included with the deterministic simulation estimations, have been obtained. In relation with path loss estimations, signal levels are within sensitivity range for almost all the locations within ICU, enabling constant communication capabilities. An ICU specific Visitor Access Control Application has been developed, based on Xbee wireless sensor nodes and a lightweight architecture implemented for the application framework, providing visitor identification and the monitoring of Boxes doors. More functionalities could be added if necessary, such as user tracking, material/waste logistics and potential extension to I/O data acquisition, such as biomedical signals or environmental signals.

The proposed ICU WSN system and application is currently being tested, with the aim of integrating the solution within a broader set of Smart Health systems at Hospital Complex of Navarra. In this sense, future work is envisaged, related with system interoperability, analysis of user dynamics or application extension with new functionalities, among others. Additionally, besides technological enhancements, further effort is required for the validation of the service, for example through usage assessment performed mainly by ICU nurses, given that the proposed system primarily alters the workload of this group of professionals—although including the other actors involved would also be of interest.

## Figures and Tables

**Table 1:** Medical equipment included in a ward of the ICU of the Hospital Complex of Navarra.

Equipment	Parameters/Techniques
Multi parameter monitor	Electrocardiogram
	Capnography
	Arterial pressure
	Pulse oximetry
	Cardiac output
	Electroencephalogram (EEG)
	Bispectral Index (derived from EEG)
Renal monitor	Slow continuous ultrafiltration
	Continuous venovenous hemodiafiltration
	Continuous venovenous hemofiltration
	Molecular Adsorbent Recirculating System (MARS)
	Plasmapheresis
Extracorporeal membrane oxygenation (ECMO) machine	Venovenous ECMO
	Venoarterial ECMO
Mechanical ventilator	Up to 60 different modes
High-flow oxygen therapy system	-
Hypothermia monitor	-

**Table 2:** Characteristics of the antennas used for measurements.

Antenna	Central Frequency	Bandwidth	Maximum Gain (dB)
FLEXI-SMA90-433	433 MHz	20 MHz	Unknown
ANT-868-CW-HWR	868 MHz	30 MHz	2
ACA-4HSRPP-2458	2.45 GHz	100 MHz	1
ACA-4HSRPP-2458	5.5 GHz	600 MHz	1

**Table 3:** 3D Ray Launching parameters.

Parameter	Value
Operation Frequency	2.41 GHz
Data rate	250 kbps
Transmitted power level	18 dBm
Antenna type	Monopole
Antenna gain (Transmitter)	1.2 dB
Antenna gain (Receiver)	1 dB
Permitted reflections	6
Launched rays angular resolution	1°
Cuboids size	0.5 m × 0.5 m × 0.5 m

**Table 4:** 3D Ray Launching parameters.

ZigBee Module	Sensitivity	Transmitted Power	Corresponding Figure
XBee-Pro	−102 dBm	10 dBm	Figure 16a
XBee-Pro	−102 dBm	2 dBm	Figure 16b
XBee	−96 dBm	−8 dBm	Figure 16c
